# Case Report: A patient with an empty sella accompanied by rare thyrotoxicosis

**DOI:** 10.3389/fendo.2025.1516538

**Published:** 2025-08-20

**Authors:** Zhaoyang Li, Kangning Han, Wenhui Yang, Bo Wu, Rufan Cao, Shiyao Chen, Xinyu Zhang, Xiaochun Han, Liangqing Guo

**Affiliations:** ^1^ The First Clinical Medical College, Shandong University of Traditional Chinese Medicine, Jinan, China; ^2^ Department of Cardiovascular, Affiliated Hospital of Shandong University of Traditional Chinese Medicine, Jinan, China; ^3^ College of Traditional Chinese Medicine, Shandong University of Traditional Chinese Medicine, Jinan, China; ^4^ College of Health, Shandong University of Traditional Chinese Medicine, Jinan, China; ^5^ Department of Endocrinology, Affiliated Hospital of Shandong University of Traditional Chinese Medicine, Jinan, China

**Keywords:** empty sella, Graves’ disease, Hashimoto thyroiditis, case report, autoimmune thyroid disease (AITD)

## Abstract

Empty sella (ES) involves herniation of the pituitary fossa, leading to pituitary flattening. While typically associated with central hypothyroidism, its co-occurrence with hyperthyroidism is rarely reported and often overlooked. We report a rare case of hyperthyroidism in a patient with ES. The patient was diagnosed with ES combined with Graves’ disease (GD) and Hashimoto’s thyroiditis (HT). Her pituitary gland appeared flattened with a depressed upper edge. The gland height was approximately 2.3 mm. Abnormal thyroid function in this case may result from the combined effects of all three conditions. A literature search on PubMed revealed a possible association between ES and autoimmune thyroid disease; however, only seven relevant studies were identified, and no standardized diagnostic or treatment protocols exist. Hyperthyroidism may mask the diagnosis of ES. In patients whose thyroid function does not normalize with conventional oral antithyroid medication, the possibility of ES should be considered. When ES is associated with primary hyperthyroidism, antithyroid drug dosages should be lower than conventional doses. Thyroid function should be monitored more frequently, and medication dosages should be adjusted promptly.

## 1 Introduction

Empty sella (ES) is a subarachnoid hernia in the pituitary fossa, which has varying degrees of influence on the pituitary gland and adjacent bone structures, and eventually leads to pituitary gland flattening. Other typical features of ES syndrome include headaches, menstrual irregularities, weight gain, and visual impairment, leading to pituitary dysfunction. These patients have endocrine disorders such as hyperprolactinemia and central hypothyroidism. In such patients, thyroid-stimulating hormone (TSH), free triiodothyronine (FT3), and free thyroxine (FT4) are often significantly reduced on endocrine examination (1).

ES is classified as either primary—attributed to arachnoid herniation—or secondary, resulting from other causes such as surgery or radiation. It is usually an asymptomatic radiological finding, with a reported prevalence ranging from 5% to 30% in imaging studies. Notably, ES is a rare cause of thyroid dysfunction, affecting the thyroid axis in approximately 1% of cases, in which central hypothyroidism typically occurs (2).

Graves’ disease (GD) and Hashimoto’s thyroiditis (HT) are common causes of thyroid dysfunction and are the two major subtypes of autoimmune thyroiditis (AITD). Although their exact etiologies remain unknown, GD and HT may coexist in the same patient. When GD and HT are present simultaneously, thyroid function tests typically show elevated FT3 and FT4 levels and suppressed TSH (3). The coexistence of ES with both GD and HT is exceptionally rare. While ES alone can lower TSH, FT3, and FT4, the presence of GD and HT complicates the thyroid function test profile, making diagnosis particularly challenging.

We report an unusual case of hyperthyroidism in a patient who failed to return to normal thyroid function after oral methimazole and was diagnosed as ES with GD combined with HT, showing partial ES on MRI. The objectives of this report are to (1) describe this rare case of ES-associated thyrotoxicosis and (2) review the literature on ES with autoimmune thyroid disease.

## 2 Case description

A 49-year-old female patient was hospitalized in the endocrinology department due to “thyroid dysfunction for more than 10 years with double vision for 1 year.” After a detailed medical history was taken, it was found that the patient had been diagnosed with hyperthyroidism more than 10 years ago due to symptoms of hyperactivity, emaciation, and abnormal thyroid function test results, and had been taking methimazole irregularly. Exophthalmos developed 2 years prior to admission but was not medically evaluated at that time. One year ago, double vision appeared, accompanied by heat intolerance and excessive sweating, and mycophenolate mofetil capsules were added.

The hospital symptoms were: double vision, photophobia with tearing, heat intolerance, excessive sweating, dry mouth, swelling and pain of the right upper limb, excessive food intake, difficulty falling asleep, frequent nocturnal awakenings, and bowel movements 2–3 times daily. The patient had a history of hepatitis B, and no history of diabetes or hypertension was found.

The medical team reviewed the patient’s thyroid function over the past 5 months and found that 5 months prior, the patient’s FT3 (11.70 pg/mL; normal value: 3.85–6.30 pg/mL) was elevated, FT4 was normal, and TSH (0.005 μIU/mL; normal value: 0.75–5.6 μIU/mL) was low. TSH receptor antibodies (TRAb) (16.40 IU/L; normal: 0.01–1.75 IU/L) were elevated. FT4 decreased for 4 months after oral administration of 10 mg/day methimazole.

After admission, laboratory examination showed that FT4 (7.81 pmol/L; normal: 12.8–21.3 pmol/L) was low, FT3 was normal, and TSH was normal but slightly below the upper limit of the reference range. Thyroid peroxidase antibodies (TPOAb) (>600 IU/mL; normal: 0–34 IU/mL), thyroglobulin antibodies (TGAb) (>4,000 IU/mL; normal: 0–115 IU/mL), and TRAb (3.09 IU/L) were all significantly elevated. Serum prolactin (PRL) was also elevated (1,075 μIU/mL; normal: 102–496 μIU/mL). Methimazole was prescribed at 5 mg/day.

**Figure 1:**
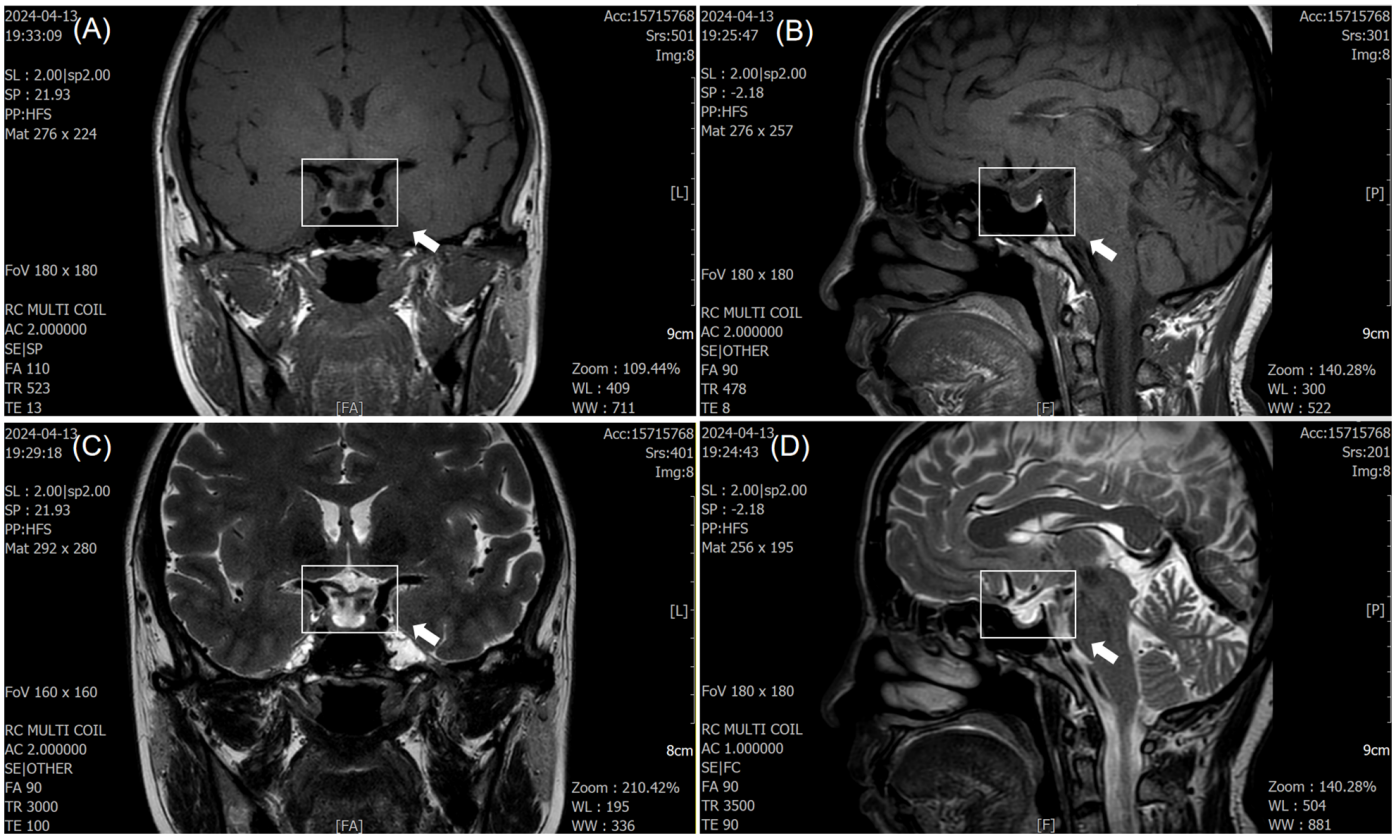
Magnetic resonance imaging (MRI) findings of empty sella syndrome. **(A, C)** T1- and T2-weighted coronal images demonstrate cerebrospinal fluid (CSF) filling the pituitary fossa (white arrow), consistent with subarachnoid herniation. **(B, D)** Sagittal images show flattening of the pituitary gland (white arrow), with the pituitary stalk positioned centrally. No optic nerve or cavernous sinus abnormalities were observed.

Due to double vision, the patient underwent cranial MRI after admission, which showed a flattened pituitary gland with partial ES. The pituitary stalk was centered, and no obvious abnormalities were observed in the optic chiasm or bilateral cavernous sinuses (**Figure 1**). Insulin-like growth factor 1, cortisol, and adrenocorticotropin levels were normal (**Table 1**).

**Table 1:** Laboratory test results.

Investigation	Result	Reference Range	Comment
Follicle stimulating hormone, mIU/mL	7.55	3.5-12.5	Normal
Luteinizing hormone, mIU/mL	5.01	2.4-12.6	Normal
Prolactin, μIu/ml	1075	102-496	High
Estradiol, Pg/mL	130.7	12.4-233	Normal
Testosterone, Pg/mL	0.42	0.084-0.481	Normal
Progesterone, Pg/mL	0.16	0.057-0.893	Normal
Insulin-like growth factor 1, ng/ml	99.18	77.2-217	Normal
Cortisol, ug/L	71	48.2-195	Normal
Adrenocorticotropic hormone, pg/ml	26.73	7.2-63.3	Normal
Anti-thyroid peroxidase antibody, IU/mL	>600	0-34	High
Anti-thyroglobulin antibody, IU/mL	>4000	0-115	High
Thyroid stimulating hormone receptor antibody, IU/L	3.09	0.01-1.75	High

**Figure 2:**
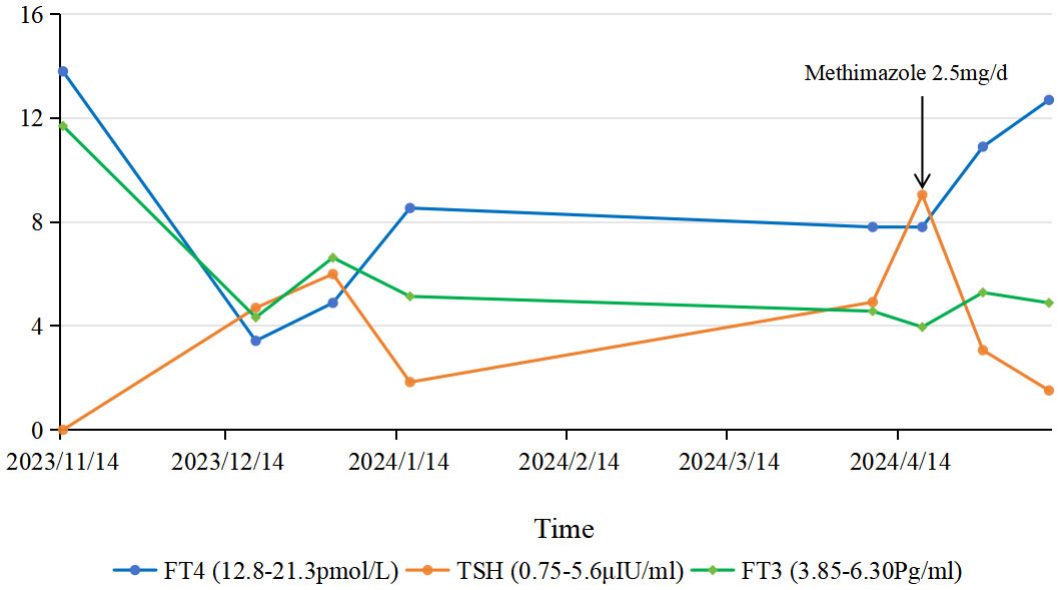
Dynamic changes in thyroid function tests. Solid lines represent patient values, and dashed lines indicate normal ranges [FT3: 3.85–6.30 pg/ml; FT4: 12.8–21.3 pmol/L; TSH: 0.75–5.6 μIU/ml]. Arrows denote key interventions: initiation of methimazole (10 mg/d) at month 0, dose reduction to 5 mg/d at month 4, and further reduction to 2.5 mg/d at month 5.

A diagnosis of ES combined with HT and GD was made based on clinical and laboratory results. The thyroid function test results were interpreted as a result of the combined influence of ES, HT, and GD. Thyroid function was reviewed 9 days after admission and showed persistently low FT4 (7.81 pmol/L) and elevated TSH. Methimazole was adjusted to 2.5 mg/day. The patient was then discharged with oral medication (**Figure 2**).

After 3 weeks, FT3 and FT4 were within normal limits, TSH was low, and PRL had returned to normal (**Supplementary Table S1**).

## 3 Method and results

We searched the literature related to ES and AITD in PubMed. The search string used was:

“empty sella”[Title/Abstract] AND (“Graves’ disease”[Title/Abstract] OR “Hashimoto thyroiditis”[Title/Abstract] OR “Hashimoto’s thyroiditis”[Title/Abstract] OR “autoimmune thyroid disease”[Title/Abstract**])**.

The filters applied were: species limited to humans, language set to English, and publication date restricted to 2010–2024.

A total of eight articles were screened. Two independent reviewers screened the titles and abstracts, followed by full-text review. Disagreements were resolved by a third reviewer. One article was excluded because ES patients were grouped with non-functioning pituitary adenoma patients.

Finally, we identified seven studies (4–10) reporting 22 patients with both ES and AITD (**Supplementary Table S2**). Key characteristics are summarized in **Supplementary Table S3**. Most patients were female (90.9%) and had ES. Hashimoto’s thyroiditis (HT) was the predominant thyroid disorder, present in 90.9% of cases.

## 4 Discussion

ES is a rare cause of thyroid dysfunction. The pathogenesis of ES is not fully understood. Primary ES syndrome may be related to congenital structural abnormalities, while secondary causes may include surgery, radiation, medication, trauma, or autoimmune disease. Although the majority of patients remain asymptomatic, a subset experiences symptoms such as headache, hypopituitarism, and blurred vision (11).

Aishah A. Ekhzaimy et al. conducted a 10-year single-center retrospective study and found that the incidence of ES was approximately 1:3.8 in males and females. They performed endocrine hormone tests on 765 patients and reported a high prevalence of coexisting endocrine abnormalities involving thyroid hormones, prolactin, LH, FSH, cortisol, testosterone, estradiol, and growth hormone (12). In a multicenter retrospective cohort study of 402 patients with ES, Giulia Carosi et al. found that pituitary hypopituitarism was common (40%), with additional endocrine abnormalities including central diabetes insipidus (1.5%) and rare cases of worsening pituitary function (3%) (13). Therefore, diagnosis and management of ES requires a multidisciplinary approach involving endocrinology, neurology, and ophthalmology.

In approximately 1% of ES cases, the thyroid axis is involved, with central hypothyroidism being the typical presentation. In a study of 43 patients with ES, S. Cannavo et al. found that only 2 patients had central hypothyroidism and 1 had primary hypothyroidism (14). The individual described in our case presented with a rare constellation of hyperthyroid symptoms. Examination revealed markedly elevated levels of TPOAb, TGAb, and TRAb, confirming the coexistence of ES with GD and HT. The patient should be distinguished from cases of primary hyperthyroidism. When thyroid function remains abnormal and is unresponsive to conventional oral drug therapy, clinicians should consider the possibility of underlying ES.

Beyond endocrine manifestations, AITD may exhibit subclinical neurological involvement. Advanced neuroimaging has revealed white matter hyperintensities, reduced gray matter volume, and altered cerebral perfusion in GD/HT patients without overt neurological symptoms (15). These changes correlate with antibody levels (TPOAb, TRAb) and may reflect autoimmune-mediated neuroinflammation (16).

Through our literature review, some researchers believe that ES is related to the incidence of AITD, though there are few relevant studies are available in PubMed. Rogelio Garcia-Centeno et al. reviewed 56 patients suspected of having ES over 20 years and found that 15 (26.78%) had AITD, suggesting that while ES affects the pituitary gland, atrophy may also occur in the thyroid gland (6). A. Yoshiiwa et al. reported a case of HT coexisting with tubular acidosis, Sjögren’s syndrome, and ES syndrome, and suggested that the onset of these diseases may be associated with autoimmunity (17). Imen Halloul et al. described a case of acromegaly complicated by ES and GD, suggesting that ES may have contributed to the development of GD (9).

Regarding the pathogenesis of ES and AITD, current research focuses on immune dysregulation involving Th cell family imbalance. Alicia Juana Klecha et al. proposed that AITD may arise from disordered Th cell immune responses, with the expression of different AITD subtypes depending on the Th1/Th2 balance (18). Agathocles Tsatsoulis described in detail that the main immune activity may promote thyroid cell apoptosis, leading to HT, whereas Th2-mediated responses may activate B lymphocytes to produce anti-TSH receptor antibodies, leading to GD (19). Silvia Martina Ferrari et al. proposed that the initial onset of AITD may be driven by Th1 activity, later transitioning to Th2 immune response (20). Further studies identified the Th1 chemokine receptor CXCR3 and its ligands CXCL9, CXCL10, and CXCL11 as key participants in AITD pathogenesis. These chemokines are secreted by damaged thyroid cells (21) and recruit Th1 lymphocytes to inflamed tissue. The recruited Th1 cells release interferon-γ (IFN-γ) and tumor necrosis factor-α (TNF-α), stimulating further secretion of Th1 chemokines and perpetuating the autoimmune loop (3).

Recent studies suggest that Th1/Th2 imbalance in AITD may be exacerbated by pituitary compression in ES, resulting in aberrant cytokine release. This aligns with our patient’s elevated TRAb/TPOAb levels and supports the hypothesis that ES may amplify thyroid autoimmunity through pituitary–immune crosstalk (22). In conclusion, the pathogenesis of AITD is closely related to Th cells and their associated immune mediators. The anterior pituitary can regulate the expression of Th cell family factors. Arjun Sharma et al. determined cytokines and chemokines in the serum of Long-Evans rats after pituitectomy by immunofluorescence and found that the concentrations of mediators were greatly increased, including IL-1α/IL-1β (10–12 times), TNF-α (19 times), Th1 cytokine, and IFN-γ (8 times) (23). Therefore, patients with ES are prone to hypophysial dysfunction due to flat pituitary morphology, and thus the chemokine and cytokine concentrations of Th cells increase, leading to immune dysfunction of Th cells, which may be the cause of ES complicated with AITD.

In this case, the main treatment was to restore thyroid function. Our patient exhibited remarkable sensitivity to methimazole. Despite a relatively low initial dose (10 mg/day) for GD, her FT4 decreased significantly within months. Following admission and confirmation of ES, an even smaller dose (5 mg/day, later reduced to 2.5 mg/day) was sufficient to normalize FT3 and FT4 within weeks. This pronounced sensitivity is likely attributable to the underlying ES-induced central hypothyroid tendency. ES reduces pituitary TSH output, diminishing the drive for thyroid hormone production. Consequently, standard doses of antithyroid drugs, aimed at suppressing hormone synthesis in a hyperstimulated gland, can lead to an exaggerated decline in thyroid hormones when this central drive is already compromised. This case strongly suggests that when ES coexists with primary hyperthyroidism, antithyroid drug doses should be initiated significantly lower than conventional recommendations and adjusted with extreme caution. Meanwhile, TSH is affected by ES, HT, and GD in many ways. Therefore, it is necessary to increase the frequency of thyroid function detection and adjust the dosage frequently.

In addition, patients have transient PRL elevation, and the most common endocrine disorder caused by ES is hyperprolactinemia. At present, it is believed that hyperprolactinemia caused by ES may have two reasons. The first reason is the “pituitary stalk effect” caused by excessive elongation of the pituitary stalk due to ES (24). The second reason is that PRL is synthesized by prolactin cells in the anterior pituitary gland and regulated by the hypothalamus. The secretion of prolactin by the hypothalamus to the pituitary gland can be divided into promotion and inhibition, mainly inhibition. Among them, prolactin inhibitory factor (PIF) inhibits the release of prolactin, and prolactin releasing factor (PRF) promotes the release of prolactin. Lacasse et al. found that prolactin concentration could be increased by injecting the dopaminergic antagonist domperidone, hence the view that dopamine is PIF (25). ES interferes with the release of dopamine in the hypothalamus, eventually leading to elevated prolactin. In addition, the increase of TSH in the recent 4 months also acts on pituitary prolactin cells and leads to the increase of prolactin.

This study has several limitations: (1) Single-case design limits generalizability. (2) Short follow-up precludes assessment of long-term outcomes. (3) Absence of cytokine profiling testing. (4) ES classification relied solely on MRI without intracranial pressure measurements or surgical confirmation. Future studies should establish multicenter registries to characterize ES-AITD interactions and investigate mechanistic cytokine profiling. Dynamic pituitary function testing should be prioritized.

## 5 Conclusion

We present a rare case of ES combined with HT and GD. ES is a rare cause of central hypothyroidism, and the diagnosis of ES is often missed in patients with hyperthyroidism. Through a literature search, evidence suggests that ES may be related to the pathogenesis of AITD, but relevant literature is limited, and the underlying mechanisms have not been comprehensively or systematically reviewed. At present, the pathogenesis is thought to involve dysregulation of Th cell family factors secreted by the pituitary gland, leading to immune disorders.

The patient in this case should be identified as having primary hyperthyroidism. When patients with hyperthyroidism fail to return their thyroid function to normal with conventional doses of oral medication, clinicians should evaluate for the possibility of ES. When ES is associated with primary hyperthyroidism, the dose of antithyroid drugs should be lower than the conventional dose, thyroid function should be tested more frequently, and the dosage should be adjusted promptly.

## Ethics approval and consent to participate

The study involving humans was approved by the Ethics Committee of the Affiliated Hospital of Shandong University of Chinese Medicine with the ethics approval number: LCYJ20241023-002 (dated October 23, 2024). Written informed consent from the patient specifically covers the publication of identifiable MRI images and clinical data presented in this case report.

## Data Availability

The original contributions presented in the study are included in the article/**Supplementary Material**. Further inquiries can be directed to the corresponding authors.

## References

[1] Ucciferro P Anastasopoulou C. Empty sella syndrome. In: StatPearls. StatPearls Publishing Copyright © 2024, StatPearls Publishing LLC, Treasure Island (FL) ineligible companies. Disclosure: Catherine Anastasopoulou declares no relevant financial relationships with ineligible companies (2024)., PMID:

[2] Kałuża B Furmanek M Domański J Żuk-Łapan A Babula E Poprawa I. The influence of pituitary morphology on the occurrence of hormonal disorders in patients with empty sella or partial empty sella. Biomedicines. (2025) 13(4):762. doi: 10.3390/biomedicines13040762, PMID: 40299333 PMC12024816

[3] Antonelli A Ferrari SM Corrado A Di Domenicantonio A Fallahi P. Autoimmune thyroid disorders. Autoimmun Rev. (2015) 14:174–80. doi: 10.1016/j.autrev.2014.10.016, PMID: 25461470

[4] Skamagas M Geer EB. Autoimmune hyperthyroidism due to secondary adrenal insufficiency: resolution with glucocorticoids. Endocr Pract. (2011) 17:85–90. doi: 10.4158/ep10069.Cr, PMID: 20841313

[5] Leães CG Rios MC Passaglia JP Pereira-Lima JF Oliveira Mda C. Autoimmune polyglandular syndrome: an unusual presentation with empty sella, premature ovarian failure, and Hashimoto’s thyroiditis associated with thyroid cancer. Gynecol Endocrinol. (2012) 28:999–1001. doi: 10.3109/09513590.2012.692222, PMID: 22686234

[6] García-Centeno R Suárez-Llanos JP Fernández-Fernández E Andía-Melero V Sánchez P Jara-Albarrán A. Empty sella and primary autoimmune hypothyroidism. Clin Exp Med. (2010) 10:129–34. doi: 10.1007/s10238-009-0071-z, PMID: 19823763

[7] Tozzi R Moramarco A Watanabe M Balena A Caputi A Gangitano E. Case report: pituitary morphology and function are preserved in female patients with idiopathic intracranial hypertension under pharmacological treatment. Front Endocrinol (Lausanne). (2020) 11:613054. doi: 10.3389/fendo.2020.613054, PMID: 33488525 PMC7819854

[8] Arpaci D Cuhaci N Saglam F Ersoy R Cakir B. Sheehan’s syndrome co-existing with Graves’ disease. Niger J Clin Pract. (2014) 17:662–5. doi: 10.4103/1119-3077.141447, PMID: 25244283

[9] Halloul I Abdelkerim AB Saad G Slim A Hasni Y Othman WB. Association of an empty sella and grave´s disease in a patient with acromegaly: a case report. Pan Afr Med J. (2021) 38:394. doi: 10.11604/pamj.2021.38.394.25034, PMID: 34381538 PMC8325439

[10] Grossi A Palma A Zanni G Novelli A Loddo S Cappa M. Multiorgan autoimmunity in a Turner syndrome patient with partial monosomy 2q and trisomy 10p. Gene. (2013) 515:439–43. doi: 10.1016/j.gene.2012.12.007, PMID: 23262341

[11] Chiloiro S Giampietro A Bianchi A De Marinis L. Empty sella syndrome: Multiple endocrine disorders. Handb Clin Neurol. (2021) 181:29–40. doi: 10.1016/b978-0-12-820683-6.00003-8, PMID: 34238465

[12] Ekhzaimy AA Mujammami M Tharkar S Alansary MA Al Otaibi D. Clinical presentation, evaluation and case management of primary empty sella syndrome: a retrospective analysis of 10-year single-center patient data. BMC Endocr Disord. (2020) 20:142. doi: 10.1186/s12902-020-00621-5, PMID: 32943019 PMC7495892

[13] Carosi G Brunetti A Mangone A Baldelli R Tresoldi A Del Sindaco G. A multicenter cohort study in patients with primary empty sella: hormonal and neuroradiological features over a long follow-up. Front Endocrinol (Lausanne). (2022) 13:925378. doi: 10.3389/fendo.2022.925378, PMID: 35813618 PMC9259926

[14] Cannavò S Curtò L Venturino M Squadrito S Almoto B Narbone MC. Abnormalities of hypothalamic-pituitary-thyroid axis in patients with primary empty sella. J Endocrinol Invest. (2002) 25:236–9. doi: 10.1007/bf03343996, PMID: 11936465

[15] Bladowska J Waliszewska-Prosół M Ejma M Sąsiadek M. The metabolic alterations within the normal appearing brain in patients with Hashimoto’s thyroiditis are correlated with hormonal changes. Metab Brain Dis. (2019) 34:53–60. doi: 10.1007/s11011-018-0318-z, PMID: 30242734 PMC6351519

[16] Waliszewska-Prosół M Bladowska J Budrewicz S Sąsiadek M Dziadkowiak E Ejma M. The evaluation of Hashimoto’s thyroiditis with event-related potentials and magnetic resonance spectroscopy and its relation to cognitive function. Sci Rep. (2021) 11:2480. doi: 10.1038/s41598-021-82281-6, PMID: 33510336 PMC7843607

[17] Yoshiiwa A Nabata T Morimoto S Sakaguchi K Yamagata H Fukuo K. A case of Hashimoto’s thyroiditis associated with renal tubular acidosis, Sjögren syndrome and empty sella syndrome. Nihon Naibunpi Gakkai Zasshi. (1992) 68:1215–23. doi: 10.1507/endocrine1927.68.11_1215, PMID: 1468597

[18] Klecha AJ Barreiro Arcos ML Frick L Genaro AM Cremaschi G. Immune-endocrine interactions in autoimmune thyroid diseases. Neuroimmunomodulation. (2008) 15:68–75. doi: 10.1159/000135626, PMID: 18667802

[19] Tsatsoulis A. The role of stress in the clinical expression of thyroid autoimmunity. Ann N Y Acad Sci. (2006) 1088:382–95. doi: 10.1196/annals.1366.015, PMID: 17192582

[20] Ferrari SM Paparo SR Ragusa F Elia G Mazzi V Patrizio A. Chemokines in thyroid autoimmunity. Best Pract Res Clin Endocrinol Metab. (2023) 37:101773. doi: 10.1016/j.beem.2023.101773, PMID: 36907786

[21] Fallahi P Elia G Ragusa F Ruffilli I Camastra S Giusti C. The aggregation between AITD with rheumatologic, or dermatologic, autoimmune diseases. Best Pract Res Clin Endocrinol Metab. (2019) 33:101372. doi: 10.1016/j.beem.2019.101372, PMID: 31932147

[22] Kinoshita R Inoue N Iwatani Y Noguchi Y Hidaka Y Watanabe M. Methylation levels of the IL10 gene in peripheral blood are related to the intractability of Graves’ disease. Clin Immunol. (2024) 263:110196. doi: 10.1016/j.clim.2024.110196, PMID: 38570004

[23] Sharma A Steven S Bosmann M. The pituitary gland prevents shock-associated death by controlling multiple inflammatory mediators. Biochem Biophys Res Commun. (2019) 509:188–93. doi: 10.1016/j.bbrc.2018.12.101, PMID: 30579593 PMC6541382

[24] Zuhur SS Kuzu I Ozturk FY Uysal E Altuntas Y. Anterior pituitary hormone deficiency in subjects with total and partial primary empty sella: do all cases need endocrinological evaluation? Turk Neurosurg. (2014) 24:374–9. doi: 10.5137/1019-5149.Jtn.8671-13.0, PMID: 24848177

[25] Lacasse P Ollier S. The dopamine antagonist domperidone increases prolactin concentration and enhances milk production in dairy cows. J Dairy Sci. (2015) 98:7856–64. doi: 10.3168/jds.2015-9865, PMID: 26298751

