# Supplementary material for: Case Report: A patient with an empty sella accompanied by rare thyrotoxicosis
Source: Front Endocrinol (Lausanne). 2025 Aug 20;16:1516538. doi: 10.3389/fendo.2025.1516538 (PMC12404976; doi:10.3389/fendo.2025.1516538)
Supplement: Supplementary file 2 [file DataSheet2.docx]

Supplementary Material

1 Supplementary Table S1: Chronological Summary of Clinical Events and Thyroid Function Parameters

| **Time Point** | **Events and Laboratory Results** |
| --- | --- |
| **>10 years ago** | Initial diagnosis of hyperthyroidism (hyperactivity, emaciation); irregular methimazole use |
| **2 years ago** | Onset of exophthalmos; no medical attention sought |
| **1 year ago** | Diplopia, heat intolerance, excessive sweating; mycophenolate mofetil capsules added |
| **5 months before admission** | **Laboratory:** ↑FT3 (11.70 Pg/ml), ↓TSH (0.005 µIU/ml), ↑TRAb (16.40 IU/L) |
| **At admission** | **Laboratory:** ↓FT4 (7.81 pmol/L), ↑TPOAb (>600 IU/ml), ↑TGAb (>4000 IU/ml), ↑PRL (1075 µIU/ml) |
| **9 days after admission** | ↓FT4 (7.81 pmol/L), ↑TSH; methimazole dosage adjusted to 2.5 mg/d |
| **3 weeks after discharge** | **Laboratory:** Normal FT3/FT4, ↓TSH, normalized PRL |

# 2 Supplementary Table S2.The list of reviewed documents

| Author | Publication Year | DOI | N°patients | Type of study |
| --- | --- | --- | --- | --- |
| Skamagas M et al. | 2011 | 10.4158/EP10069.CR | 1 | Case report |
| Leães CG et al. | 2012 | 10.3109/09513590.2012.692222 | 1 | Case report |
| García-Centeno R et al. | 2010 | 10.1007/s10238-009-0071-z | 15 | Research |
| Rossella Tozzi et al. | 2021 | 10.3389/fendo.2020.613054 | 2 | Case report |
| Arpaci D et al. | 2014 | 10.4103/1119-3077.141447 | 1 | Case report |
| Halloul I et al. | 2021 | 10.11604/pamj.2021.38.394.25034 | 1 | Case report |
| Grossi A et al. | 2013 | 10.1016/j.gene.2012.12.007 | 1 | Case report |

3 Supplementary Table S3.Summary of ES-AITD Cases from Literature Review

| Author | **Age (years)** | **Sex** | **Thyroid status** | **ES type** |
| --- | --- | --- | --- | --- |
| Skamagas M et al. | 71 | Female | GD and HT | Partially empty sella |
| Leães CG et al. | 38 | Female | HT | Empty sella |
| García-Centeno R et al. | 51.71±8.09 | Female : 13  Male : 2 | HT | Empty sella |
| Rossella Tozzi et al. | N/A | Female | HT | Empty sella |
| Arpaci D et al. | 22 | Female | GD | Empty sella |
| Halloul I et al. | 64 | Female | GD | Empty sella |
| Grossi A et al. | 3.5 | Female | HT | Partially empty sella |
